# Early malaria resurgence in pre-elimination areas in Kokap Subdistrict, Kulon Progo, Indonesia

**DOI:** 10.1186/1475-2875-13-130

**Published:** 2014-03-31

**Authors:** E Elsa Herdiana Murhandarwati, Anis Fuad, Mubarika DF Nugraheni, Sulistyawati, Mahardika A Wijayanti, Barandi S Widartono, Ting-Wu Chuang

**Affiliations:** 1 Department of Parasitology, Faculty of Medicine, Universitas Gadjah Mada, Yogyakarta, Indonesia; 2 Department of Public Health, Faculty of Medicine, Universitas Gadjah Mada, Yogyakarta, Indonesia; 3 Anthropology Department, Faculty of Arts and Humanities, Universitas Gadjah Mada, Yogyakarta, Indonesia; 4 Public Health Department, Universitas Ahmad Dahlan, Yogyakarta, Indonesia; 5 Cartography and Remote Sensing Department, Faculty of Geography, Universitas Gadjah Mada, Yogyakarta, Indonesia; 6 Department of Parasitology, Taipei Medical University, Taipei, Taiwan

**Keywords:** Malaria resurgence, Outbreak, Kokap Subdistrict, Spatial analysis, Community perspectives, Decentralization

## Abstract

**Background:**

Indonesia is among those countries committed to malaria eradication, with a continuously decreasing incidence of malaria. However, at district level the situation is different. This study presents a case of malaria resurgence Kokap Subdistrict of the Kulon Progo District in Yogyakarta Province, Java after five years of low endemicity. This study also aims to describe the community perceptions and health services delivery situation that contribute to this case.

**Methods:**

All malaria cases (2007–2011) in Kulon Progo District were stratified to annual parasite incidence (API). Two-hundred and twenty-six cases during an outbreak (May 2011 to April 2012) were geocoded by household addresses using a geographic information system (GIS) technique and clusters were identified by SaTScan software analysis (Arc GIS 10.1). Purposive random sampling was conducted on respondents living inside the clusters to identify community perceptions and behaviour related to malaria. Interviews were conducted with malaria health officers to understand the challenges of malaria surveillance and control.

**Results:**

After experiencing three consecutive years with API less than 1 per thousand, malaria in Kokap subdistrict increased almost ten times higher than API in the district level and five times higher than national API. Malaria cases were found in all five villages in 2012. One primary and two secondary malaria clusters in Hargotirto and Kalirejo villages were identified during the 2011–2012 outbreak. Most of the respondents were positively aware with malaria signs and activities of health workers to prevent malaria, although some social economic activities could not be hindered. Return transmigrants or migrant workers entering to their villages, reduced numbers of village malaria workers and a surge in malaria cases in the neighbouring district contributed to the resurgence.

**Conclusion:**

Community perception, awareness and participation could constitute a solid foundation for malaria elimination in Kokap. However, decreasing number of village malaria workers and ineffective communication between primary health centres (PHCs) within boundary areas with similar malaria problems needs attention. Decentralization policy was allegedly the reason for the less integrated malaria control between districts, especially in the cross border areas. Malaria resurgence needs attention particularly when it occurs in an area that is entering the elimination phase.

## Background

Malaria resurgence could be defined as the return to a state of equilibrium that has been disturbed by a malaria control effort [1]. Malaria resurgence is not expected to occur when an area has entered elimination phase. Cohen *et al.* reported more than 70 cases of malaria resurgence in many parts of the world, including in Indonesia. Indonesia was reported to have resurgence in 1963 and in 1973 due to funding or resource constraints, insecticide or drug resistance [1].

Malaria control in Indonesia started in 1950, mainly with DDT [2]. This effort successfully reduced malaria cases in Indonesia from an affected 30 million people in the late 1940s to around 1.1 million cases in 2009 [3]. In 2009 Indonesia declared its commitment to eliminate malaria nationally by 2030. Areas with low endemicity are encouraged to enter the elimination step earlier. Sabang municipality in Aceh is an example of an area that targeted elimination at the end of 2013 yet was previously known as one of most malarious areas in Indonesia. A tsunami disaster in 2004 indirectly helped Sabang as its malaria cases declined as health infrastructures were developed [4]. Other areas on Java Island are also expected to enter the elimination phase as their annual parasite incidence (API) was consistently less than one in 1000 people (‰) although there are some concerns, such as case finding, diagnosis, treatment, surveillance, and vector control [2].

After 2000, Indonesia showed a significant change: stable economic growth, despite a global crisis in 1997–1998, which was supported by high domestic consumption rate and decentralization. Yet, Indonesia was facing environmental problems and unavoidable climate change that might potentially trigger a resurgence. However, as Indonesia was considered to be lower middle-income country, international funding, including from the Global Fund to fight AIDS, tuberculosis and malaria was decreased.

Indonesian political and economic endorsement of decentralization influenced malaria control efforts in Indonesia. While there were several reports of malaria outbreaks, there was limited publication on the occurrence of resurgence in Indonesia.

Kulon Progo District, in particular Kokap Subdistrict, contributed to malaria outbreaks that occurred on Java Island following the economic crisis in 1997. Although this region experienced outbreaks in 1992, 1994 and 1995, the API after 1997 increased sharply and outbreaks occurred in 1998 and 1999 [5]. In 2000, the API in Kulon Progo was 85.9‰. After extra effort through *Gebrak Malaria* or “Crush Malaria” in 2000, the API had been reduced to be 83.5, 63.3, 7, 1.2, 0.4, 0.3 and 0.2‰ in 2001, 2002, 2003, 2004, 2005, 2006, and 2007, respectively [6]. In 2006, there were no high case incidence (HCI) villages, which means that malaria cases in each village were less than 5‰. Further progress was the rise in numbers of malaria-free villages, from six in 2000 to 94 in 2007 [6]. In 2010, Kokap Subdistrict was very close to malaria elimination. However, the API started to resurge in 2011 (0.32‰) and there was an outbreak in early 2012. The number of malaria cases in each village in Kokap Subdistrict, number of examined slides, origin of cases, species, API at each villages compares to Kokap Subdistrict and KulonProgo District from 2007–2012 are shown in Table 1.

**Table 1:** Distribution of total number of examined blood slides, total malaria cases, origin of cases, type of species, API by villages compares to API in Kokap subdistrict and Kulonprogo district from 2007 to 2012

			**Cases**	**Origin of cases**			**Species**			**API (‰)**		
**Year**	**Villages**	**∑ of slides**	**(n)**	**id**	**im**	**rlp**	**Pf**	**Pv**	**Mix**	**Village**	**Kokap**	**KP**
**2007**	**Kalirejo**	**900**	**2**	**1**	**1**	**0**	**0**	**2**	**0**	**0.43**		
	**Hargorejo**	**863**	**9**	**1**	**5**	**3**	**1**	**8**	**0**	**1.02**		
	**Hargomulyo**	**773**	**0**	**0**	**0**	**0**	**0**	**0**	**0**	**0**	**0.76**	**0.21**
	**Hargotirto**	**4518**	**12**	**7**	**5**	**0**	**2**	**9**	**1**	**1.49**		
	**Hargowilis**	**3202**	**6**	**1**	**5**	**0**	**0**	**6**	**0**	**0.86**		
**2008**	**Kalirejo**	**271**	**1**	**0**	**1**	**0**	**1**	**0**	**0**	**0.22**		
	**Hargorejo**	**341**	**5**	**0**	**5**	**0**	**2**	**3**	**0**	**0.57**		
	**Hargomulyo**	**145**	**0**	**0**	**0**	**0**	**0**	**0**	**0**	**0**	**0.23**	**0.16**
	**Hargotirto**	**1560**	**2**	**1**	**1**	**0**	**0**	**1**	**1**	**0.25**		
	**Hargowilis**	**881**	**1**	**0**	**1**	**0**	**0**	**1**	**0**	**0.14**		
**2009**	**Kalirejo**	**183**	**2**	**0**	**2**	**0**	**0**	**2**	**0**	**0.43**		
	**Hargorejo**	**317**	**8**	**0**	**8**	**0**	**3**	**5**	**0**	**0.91**		
	**Hargomulyo**	**81**	**0**	**0**	**0**	**0**	**0**	**0**	**0**	**0**	**0.71**	**0.29**
	**Hargotirto**	**1138**	**2**	**2**	**6**	**0**	**0**	**8**	**0**	**0.97**		
	**Hargowilis**	**870**	**9**	**0**	**9**	**0**	**0**	**9**	**0**	**1.27**		
**2010**	**Kalirejo**	**42**	**0**	**0**	**0**	**0**	**0**	**0**	**0**	**0**		
	**Hargorejo**	**152**	**1**	**0**	**1**	**0**	**1**	**0**	**0**	**0.11**		
	**Hargomulyo**	**66**	**0**	**0**	**0**	**0**	**0**	**0**	**0**	**0**	**0.29**	**0.06**
	**Hargotirto**	**875**	**9**	**3**	**6**	**0**	**0**	**8**	**1**	**1.09**		
	**Hargowilis**	**722**	**2**	**2**	**0**	**0**	**0**	**2**	**0**	**0.28**		
**2011**	**Kalirejo**	**715**	**17**	**17**	**0**	**0**	**5**	**12**	**0**	**3.67**		
	**Hargorejo**	**139**	**1**	**0**	**1**	**0**	**1**	**0**	**0**	**0.11**		
	**Hargomulyo**	**116**	**2**	**1**	**1**	**0**	**0**	**2**	**0**	**0.32**	**3.0**	**0.32**
	**Hargotirto**	**2057**	**74**	**70**	**4**	**0**	**57**	**16**	**1**	**9.43**		
	**Hargowilis**	**695**	**11**	**5**	**6**	**0**	**6**	**4**	**1**	**1.58**		
**2012**	**Kalirejo**	**1566**	**55**	**53**	**0**	**2**	**53**	**2**	**0**	**11.86**		
	**Hargorejo**	**429**	**8**	**5**	**3**	**0**	**5**	**3**	**0**	**0.91**		
	**Hargomulyo**	**130**	**1**	**1**	**0**	**0**	**0**	**1**	**0**	**0.16**	**4.8**	**0.50**
	**Hargotirto**	**5333**	**82**	**80**	**2**	**0**	**79**	**1**	**2**	**10.45**		
	**Hargowilis**	**863**	**7**	**4**	**3**	**0**	**5**	**2**	**0**	**1.01**		

API = Annual Parasite Incidence, ∑ of slides = number of examined slides, Id = indigenous cases, im = import cases, rlp = relapse cases, Pf = *Plasmodium falciparum*, Pv = *Plasmodium vivax*, mix = mixed species of *P. falciparum* and *Plasmodium vivax*, KP = Kulon Progo District. Number of examined blood slides should be at least 10% and 5% of total population in HCI and MCI areas respectively (Source: District Health Office of Kulon Progo, confirmed by Kokap I & II PHCs, 2013).

This study aims to document a case study in Kokap Subdistrict, that had been consistently defined as a low endemic area over the past five years but experienced outbreaks in 2011 and 2012. Using geographic information system (GIS) and a qualitative approach, malaria clusters during the outbreaks, and community perception towards malaria were identified. It is hypothesized whether this phenomenon is an early resurgence or just random outbreaks.

## Methods

### Study site

**Figure 1:**
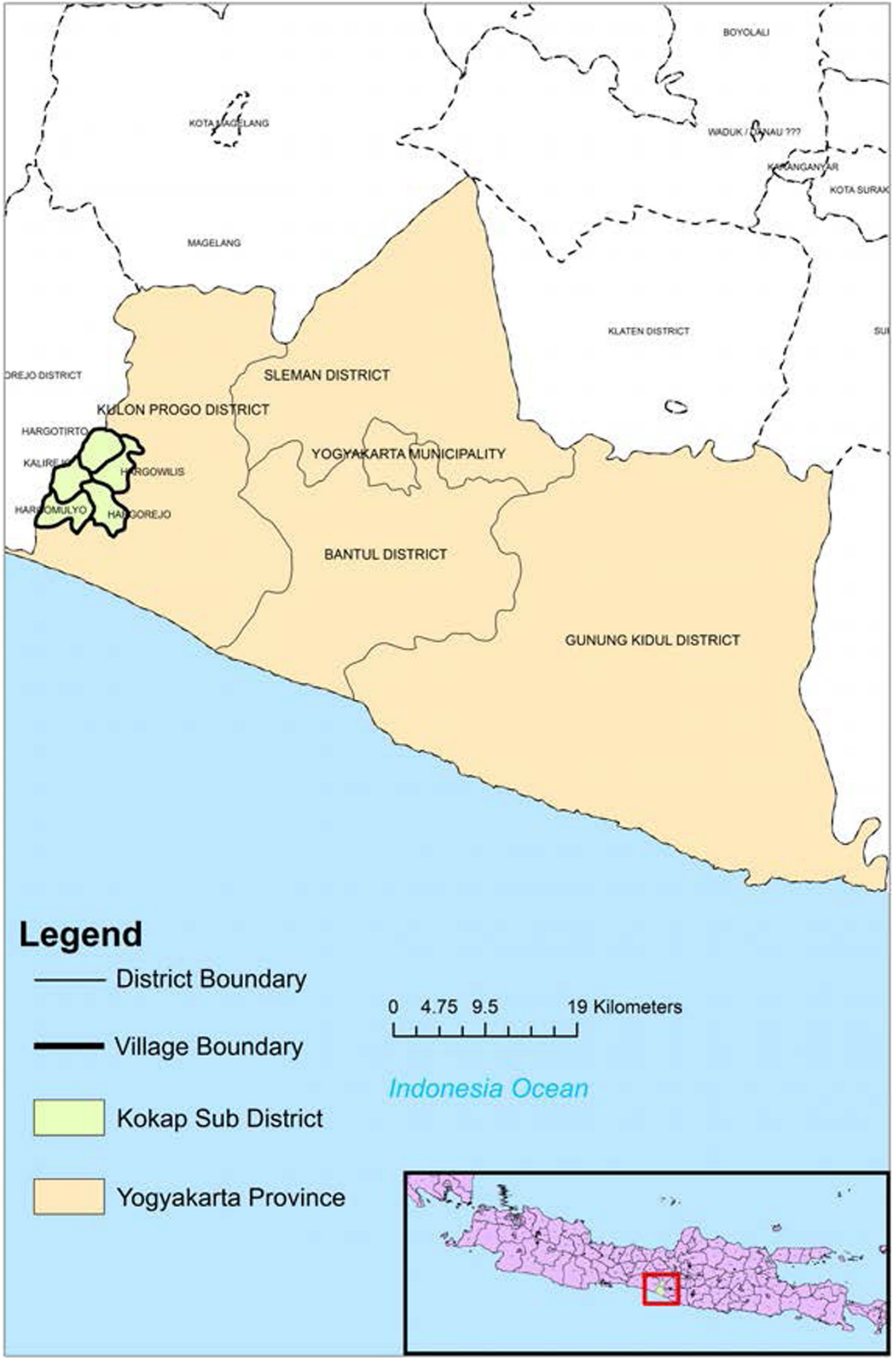
**Kokap Subdistrict (green colour) is belong to Kulon Progo District, Yogyakarta Province (orange colour) and located in Java Island Indonesia (inset).** The subdistrict is bordered with other malaria endemic subdistricts that is Girimulyo in the north which is also in Kulon Progo District, Yogyakarta Province and Bagelen & Kaligesing in the west which are belong to Purworejo District, Central Java Province. Kokap I PHC administrative area covers Kalirejo, Hargorejo and Hargomulyo villages whereas Kokap II PHC covers Hargotirto and Hargowilis villages.

A survey was conducted from May 2011 to April 2012 in Kokap, a Subdistrict in Kulonprogo, in Menoreh Hills, located between 110. 045° and 110.145° E, and 7.777° to 7. 877° S, 100 m above sea level. This subdistrict is bordered by Girimulyo Subdistrict in the north, Pengasih Subdistrict in the east, Temon Subdistrict in the south, and Bagelen and Kaligesing Subdistricts in the west, and belongs to Purworejo District (Central Java Province); it is also well known as an unstable malaria area in Java (Figure 1). Kokap Subdistrict is 73,380 sq m in area and consists of five villages. People in this subdistrict are served by two primary health centres (PHCs), Kokap I and Kokap II. Kokap I PHC covers three villages (Kalirejo, Hargorejo, Hargomulyo) whereas Kokap II PHC covers two villages (Hargotirto and Hargowilis) (Figure 1).

Kokap Subdistrict area is part of the Menoreh Hills area, located in the western part of Yogyakarta Province. The hills, together with other hills that form Yogyakarta Province, i e, Karst in the south (150–700 m above sea level), Merapi mountain in the north (80–2,911 m above sea level), and the plains area (0–80 m above sea level, located between south hill and Menoreh Hills), are located at 572 m above sea level.

**Figure 2:**
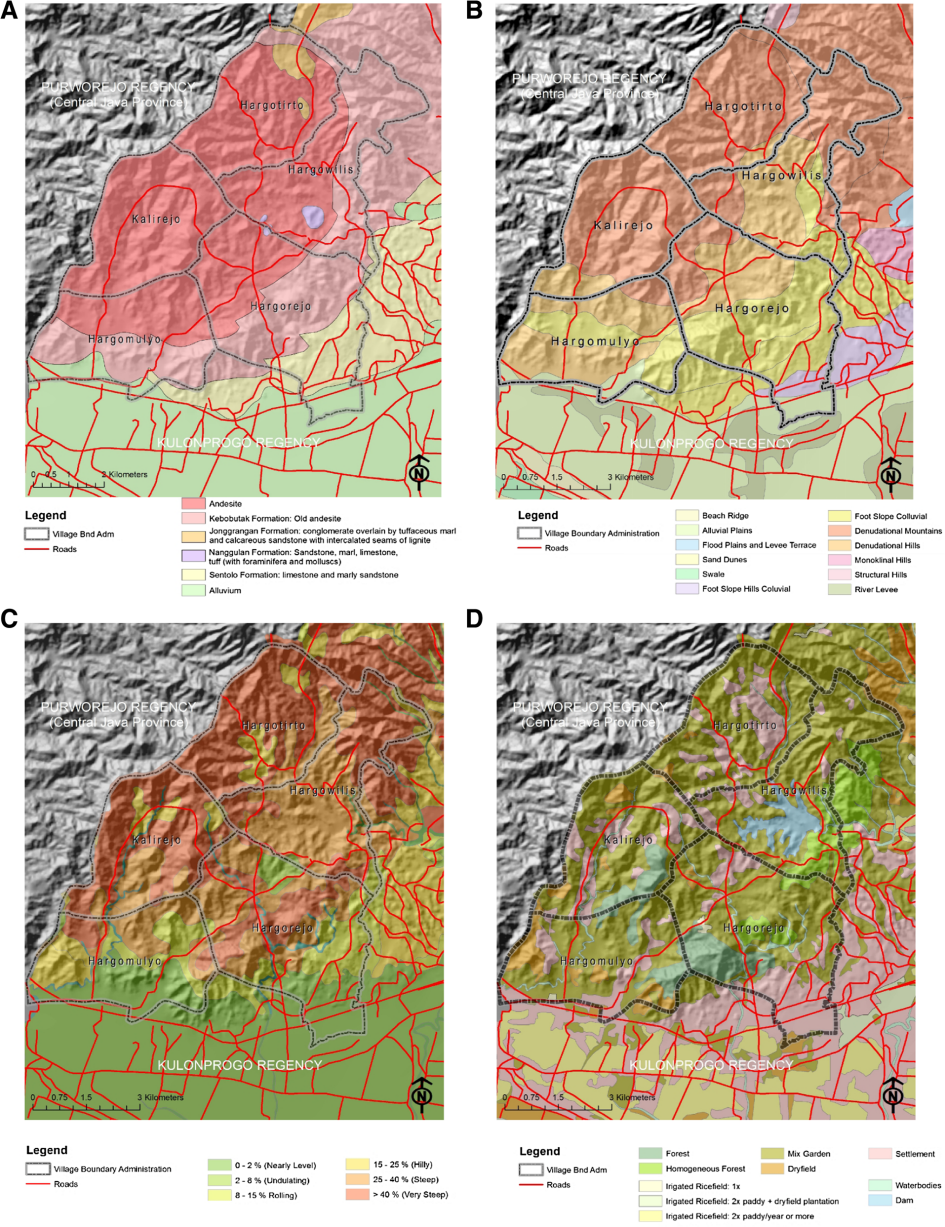
**Lithology, landform, complex slopes and land cover maps on topographic background of Kokaps subdistrict, KulonProgo, Yogyakarta in 2012.** Lithology of Kokap is dominated by andesite rock type **(A)**. Landform of Menoreh Hills is hilly to mountainous. It consists of many valleys and ridges that form many streams dominated by denudasional mountains and hills **(B)**. Complex slope of Kokap area which is dominated by very steep, steep and hilly (moderately steep) complex slope **(C)**. Land cover of Kokap, existing land cover consists mainly of forest, mixed gardens, cropland and, shrubs and bush **(D)**.

Kokap is dominated by andesite rock type (see geological map) whereas a minor part in southeastern (Sentolo formation) is arranged by aglomerat and napal (Figure 2A). Topography of the Menoreh Hills is hilly to mountainous and consists of many valleys and ridges that form many streams (Figure 2B). Kokap area is dominated by denuded mountains and hills and only a small area on the southeast slopes of the foothills is colluvial (Figure 2C). The slope of the land in Kokap area is dominated by very steep, steep and hilly (moderately steep) complex slope. Its geomorphology indicates intensive erosions causing soil layer to become thin (resulting in soil infertility) and also creating rock outcrops. Water inlets occur, particularly in the rainy season. Existing land in the study area consists mainly of forest, mixed gardens, cropland, and shrubs and bush (Figure 2D). The vegetation density might preserve temperature and humidity, particularly not fluctuating sharply, which maintains the ideal environment for mosquito breeding and resting places.

### Cross-sectional survey to map each malaria cases during malaria outbreak 2011–2012

All malaria cases (indigenous and imported) that occurred between 2007 and 2011 in Kulon Progo District were documented and stratified into low, medium or high, according to the API (<1, 1–5 and >5‰). Secondary data were obtained from Kulon Progo District Health Office. The malaria cases occurring from May 2011 to April 2012 (226 cases) were geocoded based on the household addresses using handheld GPS devices (PDA iPAQ HP connected to Compact Flash GPS/ SiRF STAR III). SaTScan 9.1 software analysis was used to identify malaria clusters using space-time permutation model (using data on location and date of cases). Arc GIS 10.1 software was used to generate the maps. Household surveys were carried out by well-trained interviewers using structured questionnaires at households with a family member confirmed with malaria. The questions addressed gender, education level, type of house wall, ventilation, floor, indoor residual spraying (IRS) in the previous year, bed net ownership, and compliance to sleep under the net, were delivered to house owners.

### In-depth interviews to obtain information related to community perceptions

Selected subjects were chosen from the cluster using snowball method until saturated information had been obtained. Twenty-four respondents from the clusters who had experienced malaria during the outbreaks or had contracted malaria in the previous three years were interviewed. A list of questions was put to the 24 respondents by two trained anthropologists. The questions included: type of occupation, types of activity from dusk to dawn, housing types, malaria perception, understanding of malaria clinical manifestations, number of malaria infections during life, having any family members who experienced malaria, having family member (s) working outside Java, suspected area where malaria infection was contracted by either respondent or family member of the respondent, actions taken when malaria infection was suspected, what is the current diagnosis at that time, actions taken against malaria either preventive or curative, local beliefs that often done for malaria treatments. Each subject was interviewed for 45 min and interviews were recorded and transcribed. Results were analysed qualitatively.

### In-depth interview to malaria health officers

Three respondents were interviewed to confirm the findings in both the cross-sectional survey and the community. The same method as above was used as above. In total there were three respondents who have been interviewed. The list of questions put to malaria health officers include: risk factor for malaria/outbreaks in their administrative area, attempts to control malaria, factors that affect the failure of malaria control. Each respondent was interviewed for 45 minutes. All interviews were recorded and transcribed and then analysed qualitatively.

This study was reviewed and approved by Institutional Review Boards for the Ethical Conduct of research on human subjects at the Universitas Gadjah Mada, Yogyakarta, Indonesia (IRB No.KE/FK/456a/EC). Informed consent was collected from participants prior to commencement of the interviews.

## Results

### Demographic characteristics of Kokap population

In 2010, the total population in Kokap was 42,264 distributed across 11,421 households [7]. Compared to other subdistricts in Kulon Progo, Kokap has less density (~0.6 people/sq m). The highest proportion of the population had primary education level. The dominant occupations were related to agriculture, trading, transportation, mining/quarrying, construction, and others. Most agriculture was in coconut or palm tree plantation. Many people depend upon their work as coconut/palm tree tappers or coconut/palm sugar makers. Coconut/sap tapper is a very risky job and is usually done twice a day, in the morning and at night. Other agriculture was paddy, cassava, peanut, corn, but with low production. According to the BPS-Statistics of Kulon Progo District [7], there are around 4,025 poor households or 33,408 poor persons living in Kokap (Table 2). This situation has forced some people to work outside the villages. In Hargotirto and Kalirejo villages, some people were reported to be transmigrants (Table 2). Working as labourers in industrial mining or logging outside Java Island was also common in Kokap Subdistrict although it is not well documented.

**Table 2:** Numbers and percentages of poor household and persons; number of transmigrant distributed in five villages in Kokap Sub District

**Villages**	**Number of poor households or persons**			**%**	**Type of transmigrants**	
	**Households**	**Persons**	**Total population**		**Number of public transmigrants**	**Number of self work transmigrants**
Hargomulyo	697	6,900	9,418	73	-	-
Hargorejo	1,049	8,601	10,768	80	-	-
Hargowilis	601	5,912	7,130	83	-	-
Kalirejo	840	4,872	5,639	86	2	7
Hargotirto	838	7,123	8,309	86	18	3
Total	4,025	33,408	41,264	81	20	10

Source: Kokap Sub District in Figures, 2010.

### Malaria endemicity 2007 to 2011 in Kulon Progo District

Based on the malaria cases stratification, the number of MCI villages in Kulonprogo District was decreasing from 2007 to 2011 (six, four, zero, two, and three villages, respectively) as well as the number of LCI villages (21, 17, six, 12, and 16 villages, respectively). HCI villages were not found from 2007 to 2010 but did appear in 2011, in one village, in Kokap Subdistrict (Figure 2).

**Figure 3:**
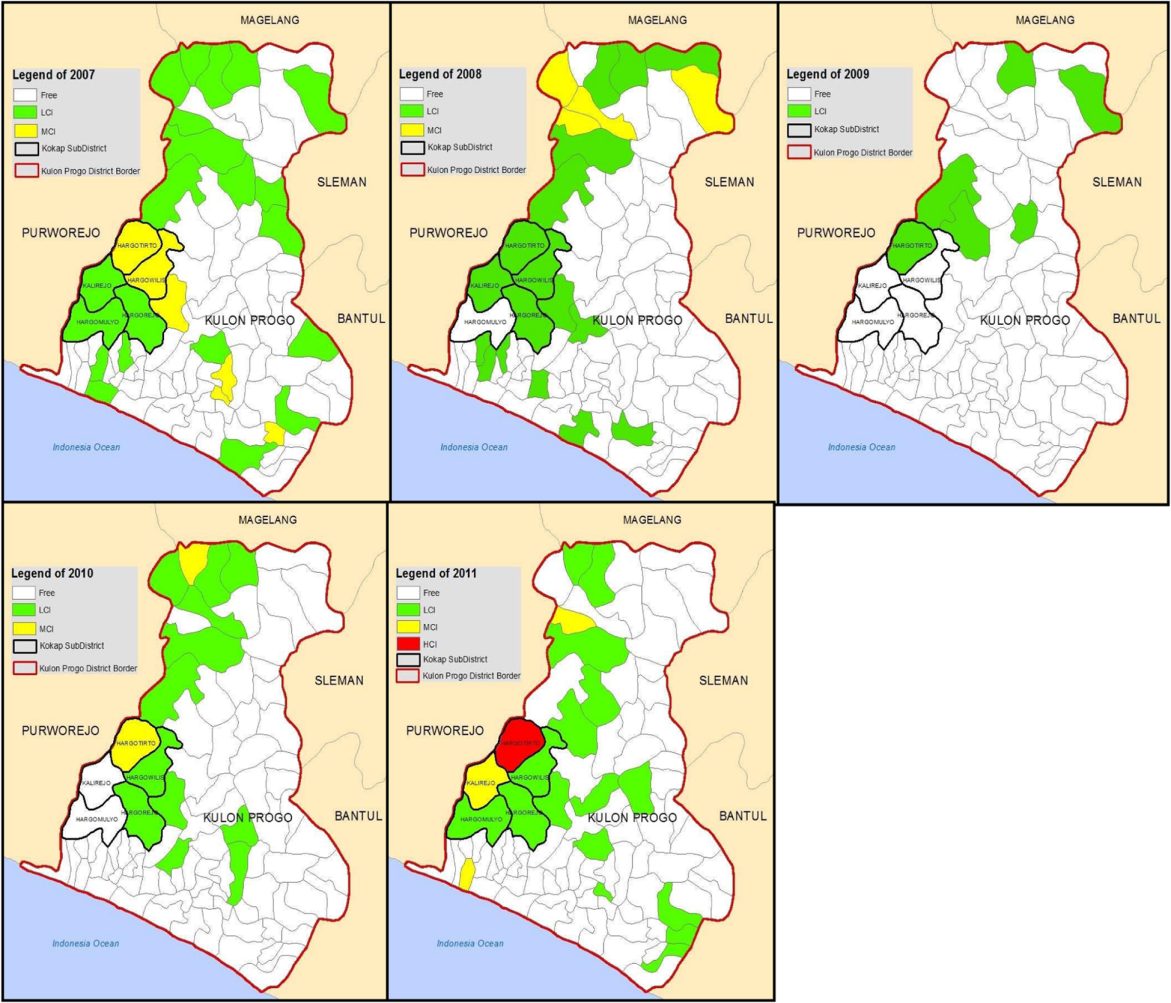
**Malaria stratification maps in Kulon Progo District including in Kokap Sub District, 2007**–**2011 (red = HCI village, yellow = MCI village, green = LCI village).**

In Kokap Subdistrict, the number of MCI village was two, zero, zero, one, and one in 2007, 2008, 2009, 2010, and 2011, respectively, whereas the number of LCI villages was around three, four, one, two, and three in the same period and no HCI village was found between 2007 and 2010. Kokap Subdistrict was almost free in 2009 with only one LCI village. However, in 2010 the LCI and MCI villages increased to two and one, respectively. In 2011, no villages in Kokap were free of malaria: Hargotirto was an HCI village, Kalirejo a MCI village and the other three villages were LCI villages (Figure 3). From the map, it can be seen that Hargotirto has never been free for malaria. *Plasmodium vivax* was the dominant species from 2007 to 2011 in Kokap I PHC and from 2007 to 2010 in Kokap II PHC. However, *Plasmodium falciparum* was more dominant in Kokap I and II PHC since 2011 and 2010 respectively (Table 1).

### Mapping of malaria cases in Kokap Subdistrict May 2011-April 2012

Two-hundred and thirty-three indigenous and imported cases were mapped using GIS. Malaria clusters were analysed using space-time scan statistics. Assuming that *Plasmodium* gametocytes develop into sporozoites in eight to 15 days in the *Anopheles* vector and need a minimum seven days to develop the clinical signs on the human host [8] thus only subsequent cases that occur within two to three weeks were included. Using those inclusion criteria, 226 cases were included.

The survey showed that a higher proportion of male patients (58%) were infected compared to females (42%). Mostly malaria infected people had no formal education (78%) and in the age group of ≥ 15 year old (77%). More patients had occupations related to agriculture (48%) than those working as labourers (6%), traders (10%) or without work (12%). The home floor types were typically soil/sand (43%) or ceramics (55%). Wall types were dominated by bamboo (35%), wood (31%), cement (30%), and brick (4%). Mostly, eaves were opened (97%). Only 16% of homes received IRS in the previous year; bed net ownership was relatively high (77%); however the compliance to sleep inside the bed nets was low (39%). Preventive actions using insecticide spray, mosquito coil or repellent were limited (Table 3).

**Table 3:** Demographic characteristics of malaria infected people during outbreaks in Kokap Sub District, Kulon Progo District (May 2011-April 2012)

**Characteristics**	**Number (percentage)**
Sex	
•Male	130 (58%)
•Female	96 (42%)
Education	
•No formal education	176 (78%)
•Primary School	41 (18%)
•Secondary School	9 (4%)
Age Group	
•A (< 5)	11 (5%)
•B (6–15)	41 (18%)
•D ≥15	174 (77%)
Occupation	
•Farmer	108 (48%)
•Labor	14 (6%)
•Trader	23 (10%)
•Not working	27 (12%)
•Others	54 (24%)
Wall types	
•Bricks	9 (4%)
•Cements	67 (30%)
•Woods	70 (31%)
•Bamboo	80 (35%)
Eaves	
•Open	219 (97%)
•Closed	7 ( 3%)
Floor types	
•Soil/sand	98 (43%)
•Ceramics/cements	125(55%)
•others	3(1%)
IRS in the last one year	
•Yes	37 (16%)
•No	189(84%)
Bednet ownership	
•Yes	175 (77%)
•No	51 (23%)
Using mosquito coils	
•Yes	9 (4%)
•No	217 (96%)
Using repellent	
•Yes	15 (7%)
•No	211(93%)
Using mosquito spray at home	
•Yes	1 (1%)
•No	225 (99%)
Compliance to sleep inside bed net	
•Yes	89 (39%)
•No	137 (61%)

Using a space-time permutation model, three significant clusters (with P-value <0.05) were identified, in Kalirejo, Hargotirto and Hargomulyo. As the primary cluster, there were one imported and 39 indigenous cases in Kalirejo, during timeframe 4 March to 5 May 2012 within radius 1.38 km. The center point of the second cluster was located at Hargomulyo village with three imported and 18 indigenous cases were found during timeframe 28 August to 15 October 2011 within radius 1.35 km. The third cluster was located at Hargotirto with two imported and 97 indigenous cases from 18 December 2011 to 14 January 2012 within radius 1.78 km.

### Malaria intervention in Kokap

Primary Health Care is the forefront health facilities to tackle malaria problem. Their staffs including clinicians, health workers, laboratory staff, health cadres, village midwives and village malaria workers were involved in malaria control. Among those, village malaria workers were the closest health persons to the community. In 2007 in Kokap I PHC, particularly in Kalirejo village, intervention included active and passive case findings, IRS (Fendona then replaced by Bistar since 2008) and surveillance migration. In 2008 to 2010, neither IRS, bed net distribution or MBS was done as number of cases was considered as small and mostly imported or relapse cases (Table 1). Only surveillance activity was still carried out at the entire period. There were eight village malaria workers in 2007 (Table 4), however, the number was reduced in 2009 and 2010 to seven and five, respectively. As Kalirejo was considered malaria free, there were two and one village malaria workers, respectively in the same years.

**Table 4:** Number of village malaria workers from 2007–2011 and its coverage in PHC I and II of Kokap Sub District

**Village**	**∑ of hamlets**	**∑ of houses**	**2007**	**2008**	**2009**	**2010**	**2011**
Kokap I PHC							
Kalirejo	9	1,060	3	3	2	1	1
Hargorejo	16	2,242	3	3	3	2	1
Hargomulyo	11	1,928	2	2	2	2	1
Kokap II PHC							
Hargowilis	13	1,609	4	4	4	3	3
Hargotirto	14	1,835	7	6	4	3	3

Source: Kokap I & II PHCs.

Similar to Kokap I PHC, intervention was particularly done in Hargotirto village included IRS, bed net distributions and MBS in 2007. Surveillance, including passive and active case finding in Kokap II PHC area, was supported by 11 village malaria workers. From 2008 to 2009, no intervention was carried out, except routine surveillance, as the relatively few malaria cases were dominated by imported cases. The reduced number of village malaria workers, i e, eight in 2009 and six in 2010, meant the surveillance that was conducted was not optimal to cover the work load.

**Figure 4:**
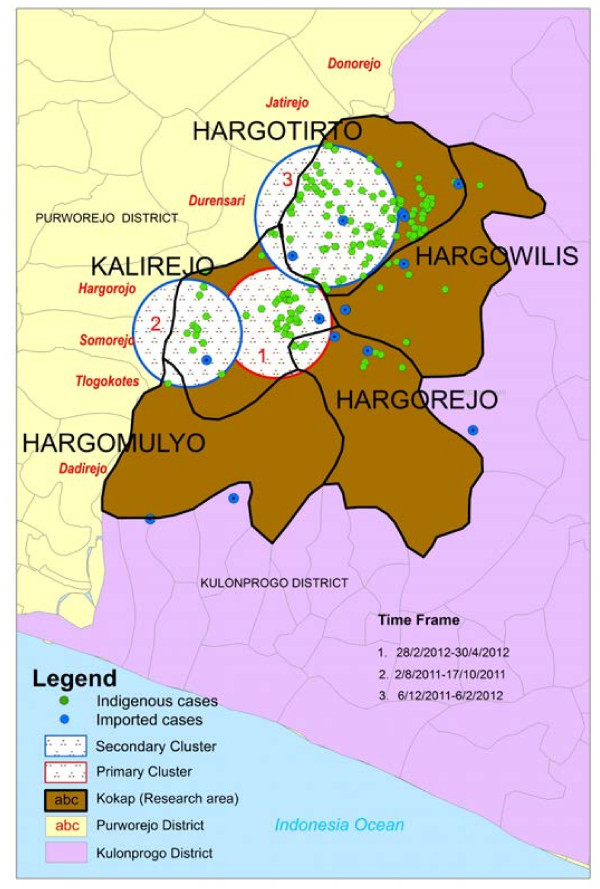
**Mapping of malaria cases in Kokap Sub District (5 villages) and its clusters during May 2011- April 2012 outbreaks.** Using Space-Time Permutation model, that considered time and locations in the analysis, three significant clusters (P-value < 0.05), shown inside the circles, were identified, i.e. Kalirejo, Hargotirto and Hargomulyo clusters.

Kalirejo and Hargotirto villages almost experiencing the similar situation with the reduction of village malaria workers. In Kalirejo for example, while in 2007 three malaria village workers covered 9 hamlets (around 1,000 households), in 2010 only one malaria village worker covered entire all 9 hamlets. Meanwhile in Hargotirto, when in 2007 seven malaria village workers covered 14 hamlets (around 2,000 households), in 2010 only three malaria village worker covered entire all 14 hamlets. This means that the workload became three times harder for village malaria worker in Kalirejo and twice harder for those in Hargotirto and as a consequence, slow down the pace of malaria finding. The increase of average number of hamlets that expected to be covered by a village malaria worker from 2007 to 2011 is shown in Figure 4.

In Kokap Subdistrict, village malaria workers were responsible to perform active case finding, ensuring the compliance of anti-malarial treatment, to carry out migration surveillance and report the finding to malaria programmer at PHC. Village malaria workers seek and visit the return immigrants after receiving the information from village leaders or the community or reversely, the return immigrants seek them to have their blood examined. The decreased of village malaria workers would indirectly decrease the number of home visits, the pace of delivering anti-malarial drug to the infected people, reduce time to monitor the compliance of anti malarial treatment and decrease the number of migration surveillance. The limited number of village malaria workers might contribute to increasing cases of malaria in 2011 and to the malaria outbreak in early 2012.

### Anthropological study on Kokap: current community response

#### Community perspective related to malaria outbreaks

A community survey was carried out in two hamlets with outbreaks in 2011–2012: Segajih, (Hargotirto village, Kokap II PHC) and Kalibuko (Kokap I PHC). In 2011, there were 24 malaria cases in Segajih hamlet, and in 2012, 18 malaria cases occurred in Kalibuko hamlet. Unstructured in-depth interviews were carried out with 12 people who had experienced malaria (in the 1997–2012 period) in each Segajih and Kalibuko hamlets and three malaria programmers at PHC.

Kokap residents were familiar with malaria and its signs, such as chills, high fever, headache, shivering, vomiting, backaches and lameness, and with the term tropical or tertian malaria, or a combination. One said that fever is not high for tertian type but is difficult to resolve with medication, while tropical type has high fever but is easily treatable. Some could even specify the nature of the parasite stages, such as gametes and the ability to transmit the malaria parasite to other people.

Some occupations and social activities need to be conducted at dusk or dawn: coconut/sap tapping for example, was carried out twice a day, i e, at 07.00-09.00 and 16.00-20.00. Village meetings, *jathilan* practice (traditional dancing), watching *jathilan* or *wayang* (traditional puppet show) performance, *pengajian* (community praying practice for Indonesian Muslim) or *njagong* (attending wedding party) were common to be hold at night-time. Both in Segajih and Kalibuko, *nira* tappers, palm/sap tappers were the dominant occupation for male respondents while the women were working as coconut sugar makers. Other occupations were nurturing Etawa goats, and cacao/clove/pepper farmers or village health cadres, gathering firewood, grazing other people’s goats or working outside Java Island as migrant workers.

The area is hilly, infertile and many water sources were drying up. Plantation, and even grass to feed cattle, were scarce. Residents blamed the situation on the decrease in numbers of livestock, thus reducing the barrier to malaria. Work as a palm coconut sap tapper is not without risk of accident. The economic burden encouraged people to work outside the region, outside Java or abroad as migrant workers. The hard socio-economic situation was also among the reason why the youth of the hamlets migrated to other districts, other Indonesian islands or outside Indonesia.

“ If we do not (work) across the ocean, there would be no progress (here)” Segajih respondent

Six of 12 respondents in Segajih hamlet had experience as labour migrants. They worked at outer Java island such as Batam, Kalimantan, Halmahera, Nusakambangan, Riau, Kalimantan or even abroad such as Malaysia. Some of them could not explain the exact address of where they worked due to their illiteracy. Some became malaria-infected while working. Residents who migrated outside Java usually worked in nickel mines, such as drilling, coal mining, palm oil or paper companies. Dormitory or housing locations and facilities provided by companies were varied, and could be in a village or close to palm plantations, forest, river, provision of which was without netting. Some male respondents who worked as sap tappers had malaria infection as a consequence of working at night, while the women who did not work outside at night believed they were infected by other family members or had experienced a malaria relapse. Respondents said that PHC advised patients to use bed nets or apply anti-mosquito lotion and not to tap after dusk. This advice was mostly complied with apart from working outside after dusk because a) sap production reaches maximum volume at night; and b) tapping before dusk did not guarantee freedom from malaria infection. Those who had been infected previously believed they did not need to be bitten as malaria (relapse, authors) can occur at any time.

However, there was an appreciated advice, i e, to provide anti-malarial for returning migrant labourers. This advice was usually followed by the labourers as it was required by the company that employed them. This information was confirmed by village leaders. One of the mining companies that belonged to a resident of Kulon Progo and employed many local people, including Segajih residents, had suffered loss due to workers suffering from malaria being unproductive. Based on this experience, the company began providing health facilities for the workers, including doctors and mosquito nets in the workplace, routine health checks and malaria treatment. It also required its employees to have malaria blood tests when they returned to Kokap: returning migrant workers in groups of around 30 workers at a time was facilitated with blood tests conducted promptly in the corporate office in the capital city, in collaboration with Kokap PHC. On receipt of the blood examination, the employee could then claim their salary. These provisions by the company might contribute to reducing malaria transmission by migrant workers.

Kalibuko respondents had different characteristics. None of them was a migrant labourer, however, most of them had family members working as migrant labourers outside Java who experienced malaria infection while working away. Whether or not infections were transmitted to family members on their return home could not be traced. The respondents had been infected by malaria while living in the village. Most respondents had houses made of bamboo. Cracks in the bamboo walls or ceilings that were commonly found in this village supported ideal malaria transmission conditions. The inaccessability of this village did not enable trucks to bring construction materials to build better houses.

Most of the respondents, confirmed by Kokap I PHC staff, were aware that the PHC had carried out several interventions, such as bed net distribution, blood survey by village malaria workers, IRS, fogging, and treating larvae instars in suspected mosquito breeding sites, such as puddles or water pools. According to Kokap I PHC staff, patient delay to visit health workers or PHC and misdiagnosis or undiagnosed malaria caused malaria transmission. A respondent in Kalibuko believed that a relative of his neighbour recently discharged from a city hospital after malaria infection, might be a source of further cases in the village. The village malaria worker confirmed this, as he had finger-pricked this patient when he suffered the fever after his return from the hospital and found parasites in the blood slide. This situation was believed to be the cause of infection of surrounding neighbours. A lack of success in controlling malaria during these outbreaks might also be caused by failure to use bed nets, damaged bed nets, wrong bed net size, non-compliance in taking anti-malarials (due to bitter taste of the drugs), or other activities at night.

Some villagers used local practices to try to cure malaria, believing that not eating a particular banana (*pisang ambon*) or sour fruits would prevent malaria relapse, and consuming bitter plants, such as extract of soursup leaves, purples mangosteen, boiled papaya leaves, ciplukan (fisalia), or brotowali (guduchi), sambiroto (creat/green chiretta/latin name: Acanthacease family) or mahoni (sky fruit) seed, would avoid or cure the disease. It was believed by doing so their blood would become bitter and mosquitoes would not like to bite. Some respondents believed that gently lashing the body with *jinggrang* leaves would avoid malaria disease.

Some respondents recalled the malaria outbreaks around 1990 when many sufferers died, and villagers held a ritual event called “*ruwatan*”, a Javanese traditional ceremony for purification to bring the community to salvation. The ceremony was initiated by villagers who had been advised by the Yogyakarta King (Sultan) who ruled at the time that it would reinforce social solidarity among villagers in their attempts to eradicate malaria:

*When malaria outbreaks occurred in 1990, our people held a* ruwatan*. Each hamlet had to prepare savory rice, rupiah coins with mountain image (100 rupiah old coins). They also had to prepare* kluwih *(young breadfruit) soups, and prayed together. All offerings were for God the Almighty as a form of prayer. In addition to fight malaria through drugs, people also prayed together. It made the Kokap people complacent in combating malaria diseases.*

Kalibuko respondent.

### PHC staff perspective of malaria outbreaks

According to PHC staff, there were several factors that influenced the outbreaks in 2011. Besides natural topography, or climate change, the surge of malaria cases in Kokap was believed to be due to the impact of uncontrolled malaria at the border area of Purworejo Province. Mobility of people between the two provinces was common and for socio-economic reasons.

Other factors were that the number of village malaria workers was not optimal for surveillance of migration, and the limitation of health cadres in reporting slides to PHC microscopists. In Kokap II PHC for example, the local government allocated four village malaria workers, while the PHC needed 11 staff although it was then anticipated by hiring six people with PHC local budget. Migration surveillance using short message service (SMS) that has been available in this District was not effective particularly when the server in District Health Office was not working. Difficult topography in Kokap area might affect the village malaria workers in bringing the slides from suspected malaria patients to microscopists at PHC laboratories, which means blood slides may be delivered in more than a day.

The PHCs stated that so far there was no problem regarding the quality of microscopic examination except that some slides delivered by village malaria workers or other health cadres did not consistently have the same quality. However, microscopic results of both PHCs showed zero error rate year by year since 2007 to 2012. A complaint from the lab staff is that sometimes the presence of *Plasmodium* parasites was uneasy to be detected by microscopic examination (low parasitaemia, authors), however after several days, the presence of gametocytes in the same patients could be then detected. This situation is causing frustration as the presence of gametocytes means that a transmission is going on.

Some activities, such as training of village health cadres and *pos penanggulangan masyarakat desa* (malaria post in high endemic and remote villages) were ceased in 2004 when malaria cases was thought to stopped occurring. Longitudinal entomological survey was not performed in Kokap II administrative area but performed at Kokap I administrative area by District Health Office during 2007–2010, although no further action was following this activity.

## Discussion

According to the District Health Office of Kulon Progo, malaria in Kulon Progo has been under control since *Gebrak Malaria* in 2001. No outbreaks have happened since then until 2011 in Hargotirto and Kalirejo. During the 2011–2012 outbreaks, three clusters were identified. One cluster was located at Hargotirto a HCI area and the other two clusters were interestingly found in MCI and LCI areas. At the end of 2012 (after this study ceased), API in both villages was still reported to be high. This outbreaks was not a random outbreak but might lead to malaria resurgence. Cohen *et al*. [1] mentioned three categories causing malaria resurgence: increasing internal potential for malaria transmission, weakening malaria control activities, and technical problems such as drug or insecticide resistance.

### Increasing internal potential for malaria transmission

Menoreh Hills is a mountainous area of Kokap, with relatively stable physical-topographic conditions that supports mosquito breeding sites for *Anopheles* by the formation of puddles throughout the year. Barcus *et al.*[9] noted that the persistence of malaria cases in this area might be worsened by the relative difficulty of removing the breeding sites of *Anopheles maculatus* and *Anopheles balabacensis*.

Some studies in Kokap Subdistrict reported potential *Anopheles* as malaria vectors. Barodji *et al*. [10] revealed that *An. maculatus* and *An. balabacencis* vectors were confirmed as malaria vectors while others were not (*Anopheles aconitus*, *Anopheles annularis*, *Anopheles barbirostris*, *Anopheles flavirostris*, *Anopheles kochi*, *Anopheles vagus*). In Hargotirto and Hargorejo, *An. balabacencis* was found as indoor-biting species while *An. maculatus* was outdoor-biting species. *Anopheles balabacencis* was ubiquitous in the mid dry season while *An. maculatus* was ubiquitous in the early dry season [10]. *Anopheles maculatus* and *An. balabacensis* species were found throughout the year, including the rainy season [11]. Thus, the presence of some *Anopheles* throughout the year confirm this region as a receptive endemic malaria area.

Hargotirto and Kalirejo is more rocky than other villages in Kokap (unpublished data), and less fertile. This condition, along with limited harvested cropland, drought, reduced number of livestock due to reduced grass as a source of animal feed, leads to poverty in the community which is dependent on agriculture. *Anopheles vagus* found in cattle was positive for circum-sporozoite protein of *P. falciparum*, and added to the list of confirmed malaria vectors in this area [12]. It is understandable that villagers blamed the situation on lack of cattle as a reason for lack of animal barrier.

Increasing numbers of the sap/coconut tapper accidents that cause permanent disability or even death, increased the economic burden on Kokap residents. This encouraged people to improve their lives by working outside the villages, including migrating to outer Java Island as labourers or transmigrants. Although transmigration in Indonesia means that families move permanently to another island that is less densely populated to seek a better life, attachment to family and homeland encouraged migrants to return to their villages, which can be problematic when the travellers return infected with malaria while working outside Java for mining, logging or farming. As most areas outside of Java are high malaria-endemic areas, the return of the people to their villages in Kokap either for religious, sociocultural events, or for visiting family will expose the villages to malaria gametocytes that are carried by the returning migrants. People movement, including migration, was considered the trigger for some malaria outbreaks. The documented record at Kokap I and II PHCs showed the estimated number of return immigrants entering Kokap SubDistrict and had their finger pricked for malaria examination (Table 5).

**Table 5:** Estimated number of return immigrants in Kokap I and II PHCs administrative area during 2007 to 2012

**Village**	**Total population**	**Number of return immigrants**					
		**2007**	**2008**	**2009**	**2010**	**2011**	**2012**
Kalirejo	5,639	25	13	2	4	5	1
Hargorejo	10,768	42	25	14	21	33	15
Hargomulyo	9,418	22	8	1	5	3	6
Hargotirto	8,309	NA	NA	NA	NA	369	411
Hargowilis	7,130	NA	NA	NA	NA	236	272

Source: Kokap I & II PHCs (NA = data is unavailable).

In Kokap study, the migrants who entered the villages were native residents of Kokap. In Brazil, massive migration of non-immune Brazilians to Amazonas and their return was initiating outbreaks in south Brazil. In Thailand, the spread of malaria risk to Thai people was due to migrant workers from neighbouring countries [13,14]. The situation might become worse particularly when malaria carriers harbour resistant *Plasmodium* strains in their blood [15].

### Weakening malaria control activities

This study observed that Kokap District villagers were aware of malaria. Most of them appreciated and complied with PHC health policy regarding malaria. The availability of documented report of return immigrants in PHCs shows the awareness from the community in dealing with malaria. The role of extended family and community to motivate these return immigrants having their blood examined by the village malaria workers or PHCs and the willingness to inform the village malaria workers regarding “ malaria potential threat” to the villages is important in controlling malaria. The experience of having *ruwatan,* a kind of genuine spirit of collectivism, as part of their local wisdom unites the community in confronting malaria. A community engagement and participation was successful in some countries in Asia and Africa regions in supporting communicable disease control [16]. Yogyakarta has a strong relationship between its King and the people with a spiritual support that was needed by devastated community. During the survey, there was community participation in dealing with malaria run by a mining company based in Wates (capital city of KulonProgo District). Both the company and local community benefitted from the practice. Augmenting social capital and sustaining community participation for elimination will be essential for elimination, especially in low malaria transmission settings, such as Vanuatu Island [17].

It is hypothesized that drastically increasing numbers of malaria cases in neighbouring areas and the decreasing number of services carried out by malaria village workers might have contributed in the resurgence (Figure 4). Data on the increasing cases in the border of Kokap and Purworejo were confirmed by respondents’ statements and by malaria health officer of Kokap PHCs and report of Purworejo District Health Office [18], while that regarding the number of malaria village workers has been confirmed by malaria health officers.

**Figure 5:**
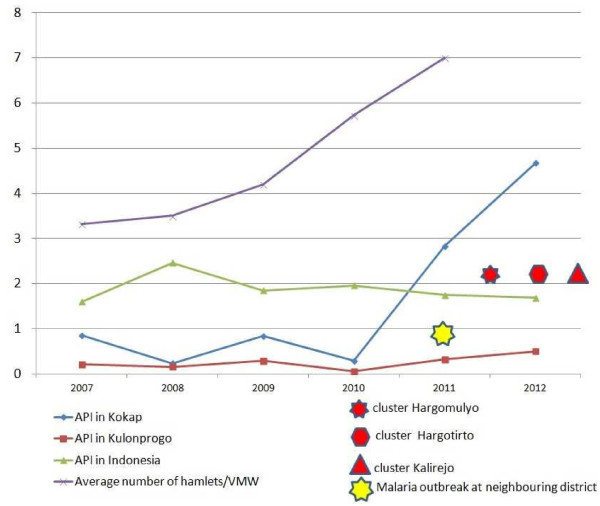
**The decreasing of national API at periode 2007–2012 was not in line with the API of Kulon Progo District and Kokap Subdistrict.** Drastic increase of Kokap API and the occurence of subsequent clusters was triggered by the increased of average number of hamlets covered by a village malaria worker as a consequence of the reduction of Village Malaria Workers number and increasing malaria cases at the border areas between two adjacent Districts.

According to Purworejo DHO report, ten villages in Kaligesing and Bagelen Subdistrict had malaria outbreaks in October 2011, of which were Donorejo and Jatirejo villages in Kaligesing Subdistrict and Hargorojo and Durensari villages in Bagelen Subdistrict that were located at the boundary area of Kokap (Figure 5) [18]. Malaria started to increase in Purworejo in 2011 with API 1.34‰, three times higher compared to 2010 (API 0.48‰). The monthly malaria incidence (MOPI) of those villages fluctuated from 5-15‰ (Somorejo), 2-10‰ (Tlogokotes), 1-3‰ (Dadirejo) from early 2011 to October 2011 and 0-6‰ (Donorejo), 0-50‰ (Jatirejo), 0-45‰ (Durensari) from early 2011 to December 2011. Those uncontrolled malaria cases at boundary areas might have been the source of malaria transmission that finally led to malaria outbreaks in Kalirejo around August to October 2011 and in Hargotirto around December 2011 to January 2012.

Serious intervention had been carried out in Kokap during the 2011 outbreaks, including IRS (994 and 2,202 housing in Kalirejo and Hargotirto villages respectively, bed net distribution (1,070 in Hargotirto village) and mass blood survey activities (377 and 448 blood slides in Kalirejo and Hargotirto respectively). Both IRS and MBS coverage achieved approximately 90% and 80% of the expected target although positive case was not found during the MBS. This intervention was fully supported by Kulon Progo District Health Office, Province Health Office and Indonesian Ministry of Health that provided technical guidance assistance and supporting facilities, and coordinated the intervention activities including infrastructure for IRS, MBS and procurement of bed nets and ACT treatment. However the subsequent surge in 2012 could not avoided. Further intervention was done i.e., IRS (207 and 3,621 in Kalirejo and Hargotirto villages respectively), bed net distributions (205 and 1,144 in Kalirejo and Hargotirto villages respectively) and MBS (423 and 2,877 blood slides in Kalirejo and Hargotirto respectively). Both IRS and MBS coverage achieved approximately 90% and 80% of the expected target, and three positive cases was found during the MBS. Malaria health officers complained of ineffective communication when integrated intervention should avoid the “ping-pong phenomena” of vector or human movements. Different districts had different planning or their own policies for malaria intervention. Administrative problems and unintegrated execution apparently played a role in providing inadequate service for malaria control as a consequence of a decentralization system.

A decentralization policy in Indonesia was aimed to increase local participation for public decision making, including health sector program. When districts area concerned with malaria as a shared problem, integrated intervention would be easier to implemented together. However, when interventions, for example mass blood surveys or IRSs, are not coordinated between border areas, efforts are wasted or remove the problem from one place to another.

This study has limitations: 1) The study was conducted after the malaria outbreaks; 2) causality of the outbreak could not be provided due to this method; 3) Centrality of the cluster based on the automated calculation could be different to the aggregated cases by administrative areas. The benefit and limitation of using space time clustering to identify centrality of outbreak should be further discussed [19] when implemented in cross border areas. However, this study is concerned with resurgence that could be established in this subdistrict, although no references that mentioned the minimal period needed to categorize an area to resurgence status is not found. With this situation continuing, concern is needed as to whether: 1) malaria resurgence in a district could drive malaria policy at province level in Indonesia where decentralization is applied? Or, 2) in case of malaria resurgence that involves cross border districts in a neighborhood provinces, stronger role of involving provinces is needed to coordinate the district. Concrete steps should be taken to address this situation otherwise malaria elimination in Java by 2015 will be impeded.

## Conclusion

Increasing numbers of malaria cases have been identified in Kokap Subdistrict, which had been facing decreasing incidence in the previous five years. Primary and secondary clusters were identified during outbreaks in 2011–2012. Community response and participation supported the elimination of malaria in Kokap. However, the return of migrants, a failure to maintain services through malaria village workers, increasing cases in adjacent areas and poor communication might have contributed to the outbreaks in 2011–2012. Caution is needed by health authorities to determine whether this is early resurgence or simply random outbreaks, which would have different implications in the field.

## Competing interests

The authors declare that they have no competing interests.

## Authors’ contributions

EEHM: conception and the design of the paper; EEHM and AF: drafting the paper critically for intellectual content; TW: involved in developing logical framework; EEHM, MN, MAW: involved in acquisition of qualitative data and interpretation; SS and BSW: involved in acquisition of GIS data and interpretation; EEHM, AF and MAW: final approval before submission. The final manuscript has been approved by all authors.
